# 4-Iodo­anilinium perchlorate

**DOI:** 10.1107/S1600536810009347

**Published:** 2010-03-20

**Authors:** Weiwei SiMa

**Affiliations:** a Ordered Matter Science Researcch Center, College of Chemistry and Chemical Engineering, Southeast University, Nanjing 210096, People’s Republic of China

## Abstract

In the crystal structure of the title compound, C_6_H_7_IN^+^·ClO_4_^−^, the ions are connected in a three-dimensional hydrogen-bonded network *via* N—H⋯O hydrogen bonds.

## Related literature

For related structures, see: Paixao *et al.* (1999 ▶); Wiedenfeld *et al.* (2004 ▶); Bendjeddou *et al.* (2003 ▶); Kapoor *et al.* (2008 ▶). For the synthetic strategy, see: Cinčić & Kaitner (2007 ▶). For bond-length data, see: Allen *et al.* (1987 ▶).
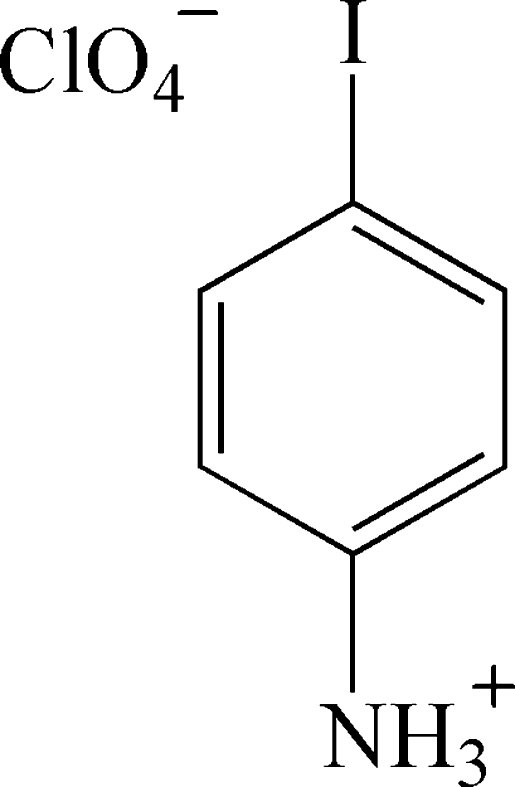


## Experimental

### 

#### Crystal data

C_6_H_7_IN^+^·ClO_4_^−^
*M* *_r_* = 319.48
Triclinic, 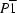

*a* = 5.105 (1) Å
*b* = 7.2445 (14) Å
*c* = 13.359 (3) Å
α = 89.47 (3)°
β = 88.74 (3)°
γ = 74.61 (3)°
*V* = 476.22 (17) Å^3^
*Z* = 2
Mo *K*α radiation
μ = 3.63 mm^−1^
*T* = 298 K
0.20 × 0.20 × 0.20 mm

#### Data collection

Rigaku SCXmini diffractometer
Absorption correction: multi-scan (*CrystalClear*; Rigaku, 2005 ▶) *T*_min_ = 0.484, *T*_max_ = 0.489
4945 measured reflections
2180 independent reflections
1956 reflections with *I* > 2σ(*I*)
*R*_int_ = 0.034

#### Refinement

*R*[*F* ^2^ > 2σ(*F* ^2^)] = 0.030
*wR*(*F* ^2^) = 0.071
*S* = 1.11
2180 reflections
119 parameters
H-atom parameters constrained
Δρ_max_ = 0.56 e Å^−3^
Δρ_min_ = −0.60 e Å^−3^

### 

Data collection: *CrystalClear* (Rigaku, 2005 ▶); cell refinement: *CrystalClear*; data reduction: *CrystalClear*; program(s) used to solve structure: *SHELXS97* (Sheldrick, 2008 ▶); program(s) used to refine structure: *SHELXL97* (Sheldrick, 2008 ▶); molecular graphics: *SHELXTL* (Sheldrick, 2008 ▶); software used to prepare material for publication: *PRPKAPPA* (Ferguson, 1999 ▶).

## Supplementary Material

Crystal structure: contains datablocks I, global. DOI: 10.1107/S1600536810009347/fi2083sup1.cif

Structure factors: contains datablocks I. DOI: 10.1107/S1600536810009347/fi2083Isup2.hkl

Additional supplementary materials:  crystallographic information; 3D view; checkCIF report

## Figures and Tables

**Table 1:** Hydrogen-bond geometry (Å, °)

*D*—H⋯*A*	*D*—H	H⋯*A*	*D*⋯*A*	*D*—H⋯*A*
N1—H1*A*⋯O2^i^	0.89	2.12	3.002 (4)	174
N1—H1*B*⋯O3^ii^	0.89	2.17	2.911 (4)	141
N1—H1*C*⋯O3^iii^	0.89	2.21	3.069 (4)	162

Symmetry codes: (i) 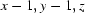; (ii) 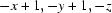; (iii) 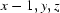.

**Fig. 1.**
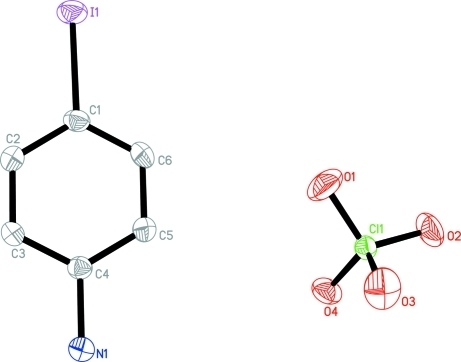
A partial packing diagram of the title compound, with the displacement ellipsoids were drawn at the 30% probability level.

**Fig. 2.**
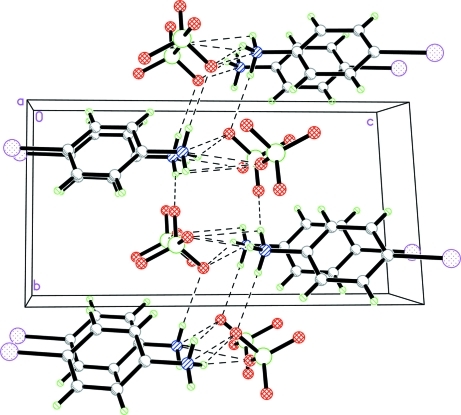
Packing diagram of the title compound viewed along the a axis. Intermolecular N—H···O hydrogen bonds are shown as dashed lines.

## supplementary crystallographic information

### Comment

To the present day a lot of structures of phenylamine perchlorate have been reported (Paixao, *et al.*, (1999); Wiedenfeld, *et al.*, (2004); Bendjeddou, *et al.*, 2003; Kapoor, *et al.*, (2008))). As part of our on-going studies on new anilinium perchloratecompounds, the crystal structure of the title compound (I) is reported herein.

The molecular structure of the title compound is shown in Figure 1. The asymmetric unit consists of one protonated 4-iodobenzenamine cation and one perchlorate anion. All bond lengths and bond angles correspond to the geometry parameters expected for atom types and the type of hybirdization (Allen *et al.*, 1987).

The ions are connected in three-dimensional hydrogen-bonded network *via* N—H···O hydrogen bonds. All ammonium group H atoms are involved in the hydrogen bonding with three O-atoms of neighbouring perchlorate anion and O-atom of carbonyl group of neighbouring cation (Figure 2).

### Experimental

The preparation of 4-iodoanilinium perchlorateis analogous to that of the compound 4-acetylanilinium perchlorate (Cinčić & Kaitner, 2007). Perchloric acid (3ml,0.16mol/L) was added to a solution of 4-iodobenzenamine (100mg) in ethanol (10ml) and the mixture was stirred for 30 min at room temperature. Colourless crystals suitable for X-ray diffraction analysis were obtained by slow evaporation of the mixed solution at room temperature after 3 days.

### Refinement

H atoms were placed at calculated position and were allowed to ride on the respective carrier atom with C—H = 0.93 Å, N—H = 0.86 Å.

### Crystal data

**Table:**

C_6_H_7_IN^+^·ClO_4_^−^	*Z* = 2
*M**_r_* = 319.48	*F*(000) = 304
Triclinic, *P*1	*D*_x_ = 2.228 Mg m^−^^3^
Hall symbol: -P 1	Mo *K*α radiation, λ = 0.71073 Å
*a* = 5.105 (1) Å	Cell parameters from 2239 reflections
*b* = 7.2445 (14) Å	θ = 2.6–27.5°
*c* = 13.359 (3) Å	µ = 3.63 mm^−^^1^
α = 89.47 (3)°	*T* = 298 K
β = 88.74 (3)°	Prism, colourless
γ = 74.61 (3)°	0.20 × 0.20 × 0.20 mm
*V* = 476.22 (17) Å^3^	

### Data collection

**Table:**

Rigaku SCXmini diffractometer	2180 independent reflections
Radiation source: fine-focus sealed tube	1956 reflections with *I* > 2σ(*I*)
graphite	*R*_int_ = 0.034
Detector resolution: 13.6612 pixels mm^-1^	θ_max_ = 27.5°, θ_min_ = 3.1°
CCD_Profile_fitting scans	*h* = −6→6
Absorption correction: multi-scan (*CrystalClear*; Rigaku, 2005)	*k* = −9→9
*T*_min_ = 0.484, *T*_max_ = 0.489	*l* = −17→17
4945 measured reflections	

### Refinement

**Table:**

Refinement on *F*^2^	Secondary atom site location: difference Fourier map
Least-squares matrix: full	Hydrogen site location: inferred from neighbouring sites
*R*[*F*^2^ > 2σ(*F*^2^)] = 0.030	H-atom parameters constrained
*wR*(*F*^2^) = 0.071	*w* = 1/[σ^2^(*F*_o_^2^) + (0.0248*P*)^2^ + 0.188*P*] where *P* = (*F*_o_^2^ + 2*F*_c_^2^)/3
*S* = 1.11	(Δ/σ)_max_ < 0.001
2180 reflections	Δρ_max_ = 0.56 e Å^−^^3^
119 parameters	Δρ_min_ = −0.60 e Å^−^^3^
0 restraints	Extinction correction: *SHELXL97* (Sheldrick, 2008), Fc^*^=kFc[1+0.001xFc^2^λ^3^/sin(2θ)]^-1/4^
Primary atom site location: structure-invariant direct methods	Extinction coefficient: 0.081 (3)

### Special details

**Table:**

Geometry. All esds (except the esd in the dihedral angle between two l.s. planes) are estimated using the full covariance matrix. The cell esds are taken into account individually in the estimation of esds in distances, angles and torsion angles; correlations between esds in cell parameters are only used when they are defined by crystal symmetry. An approximate (isotropic) treatment of cell esds is used for estimating esds involving l.s. planes.
Refinement. Refinement of F^2^ against ALL reflections. The weighted R-factor wR and goodness of fit S are based on F^2^, conventional R-factors R are based on F, with F set to zero for negative F^2^. The threshold expression of F^2^ > 2sigma(F^2^) is used only for calculating R-factors(gt) etc. and is not relevant to the choice of reflections for refinement. R-factors based on F^2^ are statistically about twice as large as those based on F, and R- factors based on ALL data will be even larger.

### Fractional atomic coordinates and isotropic or equivalent isotropic displacement parameters (Å^2^)

**Table:**

	*x*	*y*	*z*	*U*_iso_*/*U*_eq_	
I1	0.32432 (5)	0.26989 (3)	0.544100 (17)	0.05266 (14)	
N1	−0.0662 (6)	0.2407 (4)	0.1042 (2)	0.0407 (6)	
H1A	−0.1590	0.1529	0.1007	0.061*	
H1B	0.0748	0.2111	0.0615	0.061*	
H1C	−0.1738	0.3550	0.0885	0.061*	
C4	0.0315 (6)	0.2457 (4)	0.2062 (2)	0.0323 (6)	
C3	−0.0309 (7)	0.1257 (5)	0.2764 (3)	0.0436 (8)	
H3A	−0.1318	0.0410	0.2600	0.052*	
C1	0.2064 (7)	0.2578 (5)	0.3963 (2)	0.0371 (7)	
C6	0.2694 (7)	0.3764 (5)	0.3249 (3)	0.0458 (8)	
H6A	0.3722	0.4600	0.3410	0.055*	
C5	0.1789 (7)	0.3708 (5)	0.2285 (3)	0.0418 (8)	
H5A	0.2182	0.4518	0.1793	0.050*	
C2	0.0591 (8)	0.1326 (5)	0.3724 (3)	0.0482 (9)	
H2A	0.0193	0.0515	0.4214	0.058*	
Cl1	0.53128 (14)	0.77646 (10)	0.10960 (6)	0.03387 (18)	
O1	0.5785 (6)	0.6817 (5)	0.2029 (2)	0.0789 (10)	
O2	0.6164 (6)	0.9461 (4)	0.1103 (2)	0.0662 (8)	
O3	0.6814 (6)	0.6528 (4)	0.0338 (2)	0.0713 (9)	
O4	0.2516 (5)	0.8204 (4)	0.0874 (2)	0.0545 (7)	

### Atomic displacement parameters (Å^2^)

**Table:**

	*U*^11^	*U*^22^	*U*^33^	*U*^12^	*U*^13^	*U*^23^
I1	0.0751 (2)	0.04546 (18)	0.03956 (17)	−0.01822 (13)	−0.01787 (12)	−0.00395 (11)
N1	0.0449 (15)	0.0431 (15)	0.0372 (15)	−0.0169 (13)	−0.0061 (12)	0.0021 (12)
C4	0.0333 (15)	0.0308 (15)	0.0315 (15)	−0.0063 (12)	−0.0031 (12)	−0.0006 (12)
C3	0.054 (2)	0.0471 (19)	0.0392 (18)	−0.0294 (17)	−0.0100 (15)	0.0040 (15)
C1	0.0432 (17)	0.0353 (16)	0.0326 (16)	−0.0092 (14)	−0.0074 (13)	−0.0042 (13)
C6	0.058 (2)	0.0442 (19)	0.0445 (19)	−0.0295 (17)	−0.0065 (16)	−0.0043 (15)
C5	0.056 (2)	0.0386 (17)	0.0366 (17)	−0.0218 (16)	−0.0043 (15)	0.0026 (14)
C2	0.066 (2)	0.050 (2)	0.0378 (18)	−0.0307 (19)	−0.0082 (17)	0.0097 (16)
Cl1	0.0339 (4)	0.0333 (4)	0.0364 (4)	−0.0124 (3)	−0.0025 (3)	0.0032 (3)
O1	0.078 (2)	0.098 (3)	0.0568 (18)	−0.0186 (19)	−0.0113 (16)	0.0420 (18)
O2	0.0738 (18)	0.0503 (16)	0.088 (2)	−0.0392 (15)	−0.0154 (16)	0.0023 (15)
O3	0.0726 (19)	0.0593 (18)	0.078 (2)	−0.0115 (15)	0.0276 (17)	−0.0246 (16)
O4	0.0369 (13)	0.0721 (18)	0.0562 (16)	−0.0164 (12)	−0.0093 (11)	−0.0044 (14)

### Geometric parameters (Å, °)

**Table:**

I1—C1	2.086 (3)	C1—C6	1.368 (4)
N1—C4	1.465 (4)	C6—C5	1.382 (5)
N1—H1A	0.8900	C6—H6A	0.9300
N1—H1B	0.8900	C5—H5A	0.9300
N1—H1C	0.8900	C2—H2A	0.9300
C4—C5	1.361 (4)	Cl1—O2	1.408 (2)
C4—C3	1.362 (4)	Cl1—O1	1.412 (3)
C3—C2	1.377 (5)	Cl1—O4	1.416 (2)
C3—H3A	0.9300	Cl1—O3	1.426 (3)
C1—C2	1.366 (5)		
			
C4—N1—H1A	109.5	C1—C6—C5	119.4 (3)
C4—N1—H1B	109.5	C1—C6—H6A	120.3
H1A—N1—H1B	109.5	C5—C6—H6A	120.3
C4—N1—H1C	109.5	C4—C5—C6	119.3 (3)
H1A—N1—H1C	109.5	C4—C5—H5A	120.3
H1B—N1—H1C	109.5	C6—C5—H5A	120.3
C5—C4—C3	121.9 (3)	C1—C2—C3	120.6 (3)
C5—C4—N1	119.4 (3)	C1—C2—H2A	119.7
C3—C4—N1	118.7 (3)	C3—C2—H2A	119.7
C4—C3—C2	118.4 (3)	O2—Cl1—O1	110.7 (2)
C4—C3—H3A	120.8	O2—Cl1—O4	109.57 (18)
C2—C3—H3A	120.8	O1—Cl1—O4	110.10 (18)
C2—C1—C6	120.4 (3)	O2—Cl1—O3	108.95 (19)
C2—C1—I1	118.9 (2)	O1—Cl1—O3	108.8 (2)
C6—C1—I1	120.6 (2)	O4—Cl1—O3	108.73 (19)
			
C5—C4—C3—C2	0.0 (5)	N1—C4—C5—C6	179.6 (3)
N1—C4—C3—C2	−179.3 (3)	C1—C6—C5—C4	−0.8 (5)
C2—C1—C6—C5	1.1 (5)	C6—C1—C2—C3	−0.8 (6)
I1—C1—C6—C5	−177.3 (3)	I1—C1—C2—C3	177.6 (3)
C3—C4—C5—C6	0.3 (5)	C4—C3—C2—C1	0.3 (6)

### Hydrogen-bond geometry (Å, °)

**Table:**

*D*—H···*A*	*D*—H	H···*A*	*D*···*A*	*D*—H···*A*
N1—H1A···O2^i^	0.89	2.12	3.002 (4)	174
N1—H1B···O3^ii^	0.89	2.17	2.911 (4)	141
N1—H1C···O3^iii^	0.89	2.21	3.069 (4)	162

Symmetry codes: (i) *x*−1, *y*−1, *z*; (ii) −*x*+1, −*y*+1, −*z*; (iii) *x*−1, *y*, *z*.
